# The association between social media addiction and eating disturbances is mediated by muscle dysmorphia-related symptoms: a cross-sectional study in a sample of young adults

**DOI:** 10.1007/s40519-021-01232-2

**Published:** 2021-06-26

**Authors:** Claudio Imperatori, Angelo Panno, Giuseppe Alessio Carbone, Ornella Corazza, Ines Taddei, Laura Bernabei, Chiara Massullo, Elisabeth Prevete, Lorenzo Tarsitani, Massimo Pasquini, Benedetto Farina, Massimo Biondi, Francesco Saverio Bersani

**Affiliations:** 1 Cognitive and Clinical Psychology Laboratory, Department of Human Science, European University of Rome, Rome, Italy; 2 Department of Clinical, Pharmaceutical and Biological Sciences, University of Hertfordshire, Hatfield, UK; 3 Department of Medico-Surgical Sciences and Biotechnologies, Sapienza University of Rome, Latina, Italy; 4 Department of Human Neurosciences, Sapienza University of Rome, Viale dell’Università 30, 00185 Rome, Italy; 5 Mental Health Department, ASL Roma 5 Hospital, Rome, Italy

**Keywords:** Social media addiction, Eating disorders, Muscle dysmorphia, Substances use, Psychopathology

## Abstract

**Purpose:**

Although the association between problematic use of the internet and eating disorders (EDs) in young adults has been previously established, its underlying mechanisms have not been completely clarified. It has been suggested that exposure to idealized very thin and toned body images (e.g., “thinspiration” and “fitspiration” trends) on social media might lead to increased feelings of body dissatisfaction which, in turn, can represent a trigger for EDs. We have tested this hypothesis in a sample (*N* = 721) of young adults (504 females, mean age: 24.13 ± 3.70 years; range 18–34).

**Methods:**

Self-report measures investigating symptoms related to social media addiction (SMA), muscle dysmorphia (MD), and EDs were used. A mediational model analyzing the direct and indirect effects of SMA-related symptoms on ED-related symptoms through the mediating role of MD-related symptoms was performed controlling for confounding factors (e.g., socio-demographic variables, substances use, body mass index, psychopathological distress).

**Results:**

The model showed that the total effect of SMA-related symptoms on ED-related symptoms was significant (*B* = 0.213; *p* = 0.022) and that this association was mediated by MD-related symptoms (*B* = 0.083; *p* = 0.021).

**Discussion:**

Our findings support the possibility that MD-related symptoms play a relevant role in mediating the association between SMA severity and ED pathology.

**Level of evidence:**

Level III, evidence obtained from well-designed cohort or case–control analytic studies.

## Introduction

The increasing relevance of the internet in everyone’s life, and especially in the lives of adolescents and young adults, is raising interest on potential health-related sequelae associated with its use. In relation to mental health, recent studies have observed that dysfunctional and excessive use of internet, a condition often conceptualized as internet addiction or as problematic use of the internet (PUI) [1, 2], is significantly related to mental health disturbances [3, 4].

A link between PUI and mental diseases has been consistently observed in relation to eating disorders (EDs) [5]. EDs are conditions characterized by disturbed attitudes towards weight, body image, and eating, which impair physical health and psychosocial functioning [6, 7]. The Diagnostic and Statistical Manual of Mental Disorders 5th Edition (DSM5) includes a section on EDs titled “Feeding and Eating Disorders” which comprises the following diagnoses [7]: pica, rumination disorder (RD), avoidant/restrictive food intake disorder (ARFID), anorexia nervosa (AN), bulimia nervosa (BN), binge eating disorder (BED), other specified feeding or eating disorders (OSFEDs), and unspecified feeding or eating disorder (UFEDs). It is estimated that the lifetime prevalence of the main EDs (i.e., AN, BN, BED, OSFEDs) is about of 8% for women and 2% for men [8]. Furthermore, subthreshold EDs as well as ED-related symptoms (e.g., food preoccupation, binge eating, excessive dieting, body dissatisfaction, body image concerns, rigid weight-control behaviors) are widely detected with various degrees of severity in the general population, especially in young adults [9–12], as well as in patients with psychiatric diagnoses different from EDs [13–16].

In relation to the association between PUI and EDs, Hinojo-Lucena et al. recently performed two meta-analyses (involving data from over 8000 and over 5000 students) showing that both EDs (i.e., AN, BN, and BED) and ED-related symptoms (i.e., food preoccupation, loss of control eating, and dieting) were significantly higher among individuals with PUI than in those without [5].

Although the association between PUI and ED-related symptoms in young adults has been observed consistently across studies, its underlying mechanisms have not been completely clarified. In fact, the construct of PUI includes heterogeneous internet-related phenomena: as an example, disturbances related to video gaming, social networking, pornography, cybersex, and online gambling, are all tightly related to PUI but can be characterized by different underlying psychological, behavioral and biological characteristics [4, 17].

In particular, it has been suggested that one of the possible underlying mechanisms of the association between PUI and EDs is the massive exposure to idealized stylized and toned body figures on social media (SM) [5]. Specifically, studies on emerging trends among young people have been suggesting that in SM (e.g., Facebook, Instagram), there are posts, often identified with the hashtag “thinspiration”, which contribute to idealize extremely thin bodies, and that the exposure to such contents is associated with attitudes, symptoms and beliefs that characterize EDs [18–20]. Further, it has been suggested that the increasing amount of posts identified with the hashtag “fitspiration” (i.e., contents shared to promote healthy messages focused on fitness, exercise, eating styles, physical appearance, and weight control) can emphasize unrealistic body image as well as unattainable habits, thus leading to negative feelings in relation to mood, self-esteem, body image and emotional well-being [18, 21–25]. This can also contribute [26] to induce in vulnerable users symptoms related to a specific form of body dysmorphic disorder (BDD), the so called muscle dysmorphia (MD), characterized by extreme preoccupation over one’s physical appearance and muscularity and by compulsive physical exercise [16, 27].

Taking this into consideration, it is possible that excessive use of SM leads to increased levels of body dissatisfaction and MD which, in turn, can represent triggers for ED-related pathology. In the present study, we aimed at cross-sectionally exploring this phenomenon and testing this hypothesis in a sample of young adults using assessment measures specifically designed to evaluate psychopathological symptoms related to SM use, EDs and MD. Specifically we conducted a mediation analysis to examine whether MD-related symptoms mediated the association between excessive SM use and ED-related symptoms, with potential confounding factors (socio-demographic variables, substances use/misuse, body mass index [BMI] and psychopathological distress) being controlled for. We hypothesized that excessive use of SM (i) is positively associated with both MD- and ED-related symptoms, and (ii) it is positively, significantly and indirectly related to ED-related symptoms through the severity of MD-related symptoms.

## Materials and methods

### Participants and procedures

According to the sample size guidelines of Fritz and MacKinnon [28], assuming small effect sizes for the “*a*” and “*b*” paths in the mediation model, the analyses required a minimum sample size of 558 with the bootstrapping procedure to provide a statistical power of 0.80.

The present study involved 721 Italian young adults (504 females and 217 males; mean age: 24.13 ± 3.70 years; range 18–34). Due to the coronavirus disease-19 (COVID-19) outbreak, the recruitment was performed in two phases: (i) between November 2019 and March 2020 (*N* = 266, 36.9%) through a convenience sampling approach using advertising material posted around traditional community groups and using self-administered paper/pencil questionnaires; (ii) between September 2020 and December 2020 (*N* = 455, 63.1%) through an online survey shared using web-based tools (e.g., emails, mailing lists, SM, instant messaging).

All subjects voluntarily (i.e., they did not receive payment or other compensation) and anonymously participated to the present research and gave their written informed consent to take part to the study. Inclusion criteria were: (i) age between 18 and 34 years, (ii) good ability to understand written Italian, (iii) for the online survey, the correct response to one item of attentional quality check, and (iv) the provision of written consent.

### Measures

Information on socio-demographic and clinical variables [i.e., age, sex, body mass index (BMI, job status, education level, marital status, tobacco and illicit drugs use in the last 12 months] was collected. Subjects were asked to complete the following self-report measures: the Bergen Social Media Addiction Scale [BSMAS; 29], the Eating Attitudes Test-26 [EAT-26; 30], the Muscle Dysmorphic Disorder Inventory [MDDI; 31], the Brief Symptom Inventory [BSI; 32] and the Cut-Annoyed-Guilty-Eye (CAGE) questionnaire [33].

The BSMAS is a 6-item self-administered questionnaire assessing, in the last 12 months, addiction-like symptoms (i.e., salience, mood modification, tolerance, withdrawal, conflict, and relapse) in relation to excessive and compulsive use of SM (e.g., Facebook, Instagram, etc.). Items are rated on a 5-point Likert scale (from 1 = “very rarely” to 5 = “very often”) with higher scores reflecting higher levels of symptoms related to social media addiction (SMA). A cut-off of ≥ 19 has been used to screen problematic social media use (PSMU) [34]. In the present study the Italian version of the BSMAS [35] was used and the Cronbach’s *α* was 0.85.

The EAT-26 is a 26-item self-report questionnaire commonly used to investigate symptoms and concerns characteristic of EDs [36], such as dieting and food preoccupation. Participants were asked to judge each items according to 6-point Likert scale (from “never” = 0 to “always” = 3) with total scores ranging from 0 to 78. Higher scores reflect higher ED-related symptoms, and a cut-off of ≥ 20 is widely used as the threshold value for clinically significant ED-related pathology [37]. The Italian validated version of the EAT-26 used in this study has showed good psychometric properties [38]. Cronbach’s alpha in our sample was 0.93 for the EAT-26 total score.

The MDDI is one of the most used tools for assessing MD [39]. It is a multi-dimensional self‐report measure composed by 13 items and rated on a 5-point Likert-type scale (from 1 = “never” to 5 = “always”) investigating three MD core dimensions, confirmed through factor analyses [39]: (i) drive for size (e.g., thoughts of being thinner and/or the desire to increase size and strength), (ii) appearance intolerance (e.g., negative beliefs about one’s body), and (iii) functional impairment (e.g., excessive and compulsive exercise). A total score can be derived by the sum of the subscales. Higher values reflect higher MD-related symptoms, and a cut-off point of 39 has been proposed and used in previous reports to identify clinically relevant MD [40, 41]. Although the psychometric properties of the MDDI were most investigated in male samples, it has been recently reported [40] that the three-factor structure of the MDDI was independent of sex with no statistically significant difference in the MDDI total score. In the present study, the Italian version of the MDDI [42] was used and the Cronbach’s *α* was 0.78 for the MDDI total score.

The BSI [32] is the short version of the Symptom Checklist-90R [43] and it is composed by 53 items rated on a 5-point Likert scale (0–4) assessing nine primary symptom dimensions: depression, somatization, obsessive–compulsive, interpersonal sensitivity, anxiety, hostility, phobic anxiety, paranoid ideation, and psychoticism. The BSI also provides a global severity index (GSI) which is designed to measure overall psychopathological distress. Higher scores indicate more severe self-reported symptoms. As recommended [32] and according to previous reports e.g., [44–46], in the present study the cutoff point of 63 T score on the GSI or in two primary dimensions was used to identify subjects with clinically relevant level of psychopathological distress. We used a previously validated Italian version of this scale [47] and the Cronbach’s alpha in the present sample was 0.97 for the GSI.

The CAGE questionnaire is composed by 4 dichotomous (0 = “no”; 1 = “yes”) items and it is designed as screening tool for the detection of problematic alcohol use (PAU). The total score can range from 0 to 4, with higher values reflecting more severe problematic patterns of alcohol use. A cut-off of ≥ 2 is commonly used in the literature [48, 49] to screen PAU. In the present study, we used the Italian adaptation of the CAGE [33], and the Cronbach’s α was 0.64 for the CAGE total score.

### Statistical analyses

All analyses were performed using the SPSS (18.0) statistical package (IBM, Armonk, NY, USA). The association among variables was assessed using Spearman’s *ρ* correlation coefficients. For sensitivity analyses, differences between individuals with and without PSMU (i.e., BSMAS ≥ 19) were also computed using two-way Chi-squared (*χ*^2^) and Mann–Whitney *U* test for dichotomous and dimensional measures, respectively. Analysis-appropriate effect sizes (Cohen’s *d* and Cramer’s *V,* respectively, for *U* and *χ*^2^ tests) were computed [50] and converted to *r* values [51]. Non-parametric tests were chosen as several of the considered variables were not normally distributed (Shapiro–Wilk test, *p* < 0.05).

To determine whether the relationship between SMA- and ED-related symptoms was mediated by MD severity, a mediation model using the Model 4 of the SPSS Macro Process [52] with 5000 bootstrap samples was performed. In the present study, we tested a model in which BSMAS total score (i.e., SMA-related symptoms) was the independent variable, EAT-26 total score (i.e., ED-related symptoms) was the dependent variable, and MDDI total score (MD-related symptoms) was the mediator. Potentials cofounding sociodemographic (i.e., sex, age, educational level, marital and job status) and clinical (i.e., BMI, problematic alcohol use, tobacco and illicit drug use, and clinically relevant psychopathological distress) variables were also included in the model as covariates. Furthermore, due to the different recruitment method and taking into account the impact of COVID-19 pandemic on psychopathology (including potential impact on substance and behavioral addictions) [53–55], the compilation modality (i.e., paper/pencil vs online questionnaires) was also included as a covariate.

Although in the mediational models, the relations between variables are considered to be causal, for cross-sectional studies, the mediation strategies should be considered as a type of variance partitioning, which can be useful when investigating whether the relation between two variables is reduced when a mediating variable is considered [56]. Thus, it is important to note that the adopted statistical design is correlational in nature, which precludes a definitive causal interpretation of the association between these variables. According to Baron and Kenny [57], we reported the results from different three models. In the first model (path *a*) we showed the direct effect of SMA-related symptoms on MD-related symptoms. In the second model (path *c*′) we reported the direct effect of SMA-related symptoms on ED-related symptoms. In the third and last model (path *c*), we showed the total effect, i.e., the sum of the direct and indirect effects, of SMA-related symptoms on ED-related symptoms.

### Ethics

The present study was approved by the Ethics committee of the Department of Human Neurosciences of Sapienza University of Rome and was performed in accordance with the Helsinki Declaration criteria.

## Results

In the present sample, there were 154 subjects (21.4%) who met the criteria for PSMU, 120 (16.6%) who met the criteria for clinically relevant ED-related symptoms, and 26 (3.6%) who met the criteria for clinically relevant MD. Finally, there were 170 participants (23.6%) who met the criteria for clinically relevant psychopathological distress, 94 (13.0%) who met the criteria for PAU. Detailed clinical and socio-demographic characteristics of the sample are reported in Table 1.

**Table 1:** Descriptive statistics for the sample (*N* = 721)

Variables	
Age, M ± SD	24.13 ± 3.70
Females, *N* (%)	504 (69.9)
Occupation	
Employed, *N* (%)	195 (27.1)
Unemployed, *N* (%)	47 (6.5)
Students, *N* (%)	479 (66.4)
Married or living with partner, *N* (%)	84 (11.7)
Educational level > 13 years, *N* (%)	316 (43.8)
Tobacco use in the last 12 months, *N* (%)	311 (43.1)
Substance use in the last 12 months*, *N* (%)	161 (22.3)
CAGE total score, M ± SD	0.45 ± 0.86
CAGE ≥ 2, *N* (%)	94 (13.0)
Self-reported BMI, M ± SD	22.10 ± 3.28
BMI < 18.5 kg/m^2^, *N* (%)	77 (10.7)
BMI between 18.50 and 24.99 kg/m^2^, *N* (%)	534 (74.1)
BMI between 25 and 29.9 kg/m^2^, *N* (%)	95 (13.2)
BMI ≥ 30 kg/m^2^, *N* (%)	15 (2.1)
BSMAS total score, M ± SD	14.21 ± 5.47
BSMAS ≥ 19, *N* (%)	154 (21.4)
EAT-26 total score, M ± SD	10.08 ± 12.93
EAT-26 ≥ 20, *N* (%)	120 (16.6)
MDDI total score, M ± SD	23.33 ± 7.65
MDDI > 39, *N* (%)	26 (3.6)
BSI-GSI, M ± SD	0.97 ± 0.70
BSI cut-off**, *N* (%)	170 (23.6)

*M* mean, *SD* standard deviation, *CAGE* Cut-Annoyed-Guilty-Eye (CAGE) questionnaire, *BMI* body mass index, *BSMAS* Bergen Social Media Addiction Scale, *MDDI* Muscle Dysmorphic Disorder Inventory, *EAT-26* Eating Attitudes Test-26, *BSI-GSI* Brief Symptom Inventory-Global severity Index

*Number of individual who reported that the most relevantly use psychoactive substance in the previous years was one of the following: cannabis, cocaine, heroin, hallucinogens, amphetamines, tranquillizers, other substances different from alcohol, nicotine and caffeine

**63 T score on the GSI or in two primary BSI dimensions

SMA-related symptoms were positively associated with MD-related symptoms (*ρ* = 0.138; *p* < 0.001), EDs-related symptoms (*ρ* = 0.179; *p* < 0.001), psychopathological distress (*ρ* = 0.430; *p* < 0.001) and PAU severity (*ρ* = 0.105; *p* = 0.005). A similar pattern of correlations was detected for both MDDI and EAT-26 total score. SMA-related symptoms were also negatively associated with both age (*ρ* = − 0.163; *p* < 0.001) and BMI (*ρ* = − 0.164; *p* < 0.001). Detailed correlations among the variables are reported in Table 2. Differences between individuals with and without PSMU are reported in Table 3.

**Table 2:** Values of Spearman’s *ρ* correlation coefficient among variables in all samples (*N* = 721)

	1	2	3	4	5	6	7
1. BSMAS total score	–						
2. MDDI total score	0.138***	–					
3. EAT-26 total score	0.179***	0.459***	–				
4. BSI-GSI	0.430***	0.354***	0.382***	–			
5. CAGE total score	0.105**	0.220***	0.180***	0.205***	–		
6. Age	− 0.163***	− 0.068	− 0.090*	− 0.145***	− 0.023	–	
7. Self-reported BMI	− 0.164***	0.048	− 0.011	− 0.116**	0.028	0.165***	–

Significant correlations are indicated by asterisk (*)

*BSMAS* Bergen Social Media Addiction Scale, *MDDI* Muscle Dysmorphic Disorder Inventory, *EAT-26* Eating Attitudes Test-26, *BSI-GSI* Brief Symptom Inventory-Global severity Index, *BMI* body mass index, *CAGE* Cut-Annoyed-Guilty-Eye (CAGE) questionnaire

*p < 0.05; **p < 0.01; ***p < 0.001

**Table 3:** Differences between participants with and without problematic social media use according BSMAS ≥ 19

Variables	PSMU− *N* = 567	PSMU+ *N* = 154	Test	*p*	Effect size, *r*
Younger (18–24) young adults, *N* (%)	335 (59.1%)	106 (68.8%)	*χ*^2^ = 4.845	**0.028**	0.082
Age, M ± SD	24.33 ± 3.75	23.40 ± 3.42	*U* = 37260.0	**0.005**	0.104
Females, *N* (%)	382 (67.4%)	122 (79.2%)	*χ*^2^ = 8.081	**0.004**	0.106
Students, *N* (%)	365 (64.4%)	114 (74.0%)	*χ*^2^ = 5.060	**0.024**	0.084
Employed, *N* (%)	166 (29.3%)	29 (18.8%)	*χ*^2^ = 6.687	**0.010**	0.096
Educational level > 13 years, *N* (%)	261 (46.0%)	55 (35.7%)	*χ*^2^ = 5.237	**0.022**	0.085
BMI < 18.5 kg/m^2^, *N* (%)	53 (9.3%)	24 (15.6%)	*χ*^2^ = 7.065	0.070	0.172
BMI between 18.50 and 24.99 kg/m^2^, *N* (%)	422 (74.4%)	112 (72.7%)			
BMI between 25 and 29.9 kg/m^2^, *N* (%)	78 (13.8%)	17 (11.0%)			
BMI ≥ 30 kg/m^2^, *N* (%)	14 (2.5%)	1 (0.6%)			
BMI, M ± SD	22.30 ± 3.30	21.39 ± 3.13	*U* = 36207.0	< **0.001**	0.121
Married or living with partner, *N* (%)	71 (12.5%)	13 (8.4%)	*χ*^2^ = 1.959	0.162	0.052
Tobacco use (last 12 months), *N* (%)	253 (44.6%)	58 (37.7%)	*χ*^2^ = 2.391	0.122	0.058
Substances use (last 12 months), *N* (%)	135 (22.8%)	26 (16.9%)	*χ*^2^ = 3.350	0.067	0.068
CAGE ≥ 2, *N* (%)	70 (12.3%)	24 (15.6%)	*χ*^2^ = 1.120	0.290	0.039
CAGE total, M ± SD	0.42 ± 0.82	0.58 ± 0.99	*U* = 40225.5	0.053	0.056
EAT-26 ≥ 20, *N* (%)	81 (14.3%)	39 (25.3%)	*χ*^2^ = 10.638	**0.001**	0.121
EAT-26 total score, M ± SD	9.15 ± 12.23	13.49 ± 14.77	*U* = 36653.5	**0.002**	0.114
MDDI > 39, *N* (%)	18 (3.2%)	8 (5.2%)	*χ*^2^ = 1.422	0.233	0.044
MDDI total score, M ± SD	22.92 ± 7.47	24.84 ± 8.13	*U* = 37611.0	**0.008**	0.098
BSI cut-off, *N* (%)	99 (17.5%)	71 (46.1%)	*χ*^2^ = 55.143	< **0.001**	0.277
BSI-GSI, M ± SD	0.84 ± 0.63	1.45 ± 0.71	*U* = 21901.5	< **0.001**	0.354

Bold values represent* p *< 0.05

Original, *BSMAS* Bergen Social Media Addiction Scale, *PSMU* problematic social media use, *CAGE* Cut-Annoyed-Guilty-Eye (CAGE) questionnaire, *MDDI* Muscle Dysmorphic Disorder Inventory, *EAT-26* Eating Attitudes Test-26, *BSI-GSI* Brief Symptom Inventory-Global severity Index

**Fig. 1:**
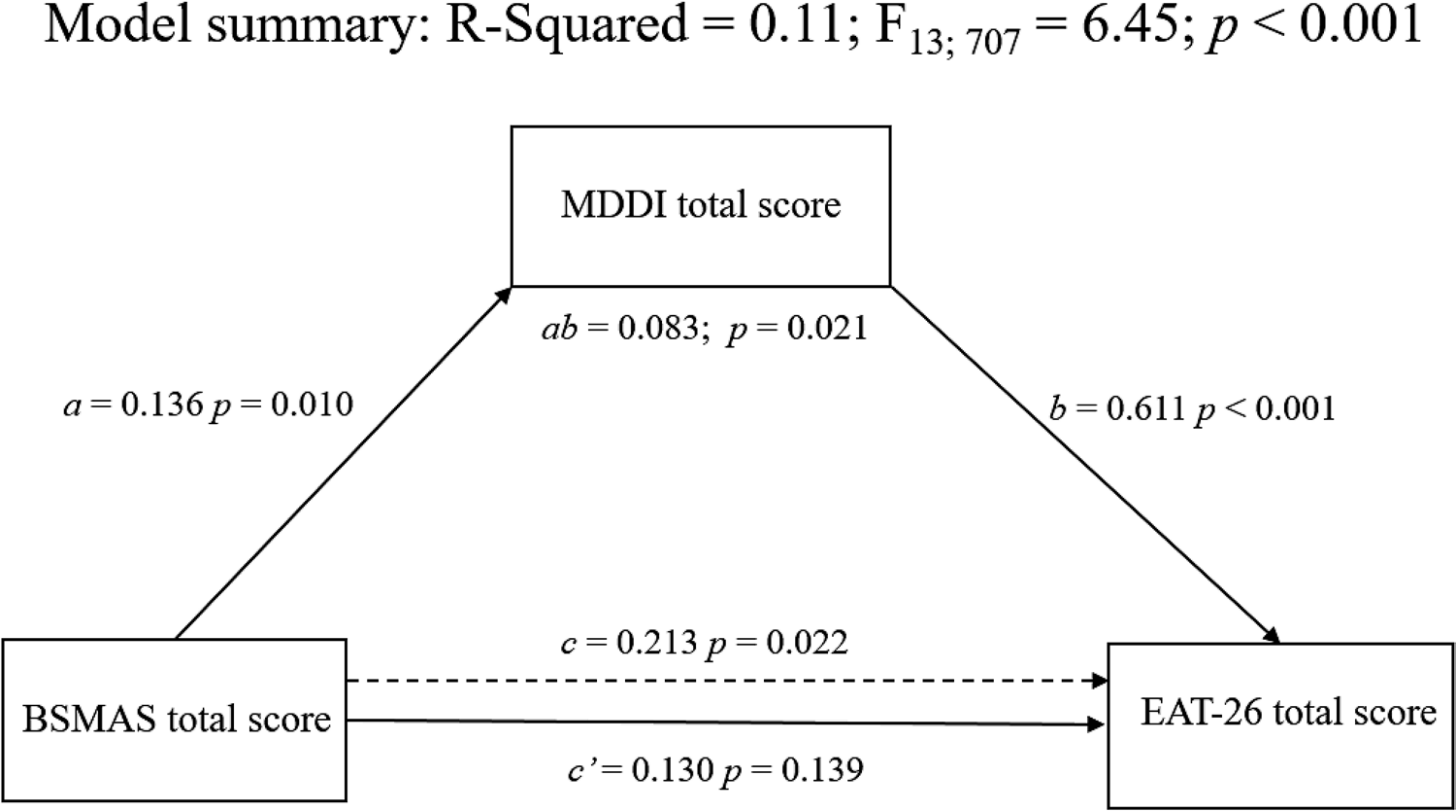
Graphical representation of the results from the mediation model. The reported estimates were obtained controlling for potentially competing factors (i.e., compilation modality, socio-demographic variables, tobacco use, problematic alcohol use, substances use, self-reported body mass index, and clinically relevant psychopathological distress). *BSMAS* The Bergen Social Media Addiction Scale, *EAT-26* Eating Attitudes Test, *MDDI* Muscle Dysmorphia Disorder Inventory

Results of the mediational model are reported in Table 4 and graphically showed in Fig. 1. Specifically, the total effect was positive and significant (*R*^2^ = 0.11; *F*_13; 707_ = 6.45; *p* < 0.001), indicating that higher SMA-related symptoms were associated to higher ED-related symptoms (*B* = 0.213; *p* = 0.022). Moreover, the effect of SMA-related symptoms on MD-related symptoms was positive and significant (*B* = 0.136; *p* = 0.010). In turn, MD-related symptoms were positively related to ED-related symptoms (*B* = 0.611; *p* < 0.001). Lastly, (i) the indirect effect was significant [*B* = 0.083, SE = 0.036, *p* = 0.021 (CI 0.016; 0.159)], thus confirming our mediation hypothesis, and (ii) the direct effect of SMA-related symptoms on ED-related symptoms was not significant (*B* = 0.130; *p* = 0.139), thus suggesting that the association between SMA-related symptoms and ED-related symptoms was mediated by MD-related symptoms.

**Table 4:** Results of the mediation models

	Muscle dysmorphia severity			Eating disorder-related symptoms					
	Model 1 (path *a*)			Model 2 (path *c*′)			Model 3 (path *c*)		
	*B*	SE	95% CI	*B*	SE	95% CI	*B*	SE	95% CI
BSMAS total score	0.136*	0.052	[0.033; 0.239]	0.130	0.088	[− 0.042; 0.301]	0.213*	0.093	[0.031; 0.395]
MDDI total score	–	–	–	0.611***	0.063	[0.488; 0.733]	–	–	–
Age	− 0.025	0.099	[− 0.219; 0.169]	− 0.056	0.164	[− 0.379; 0.266]	− 0.072	0.175	[− 0.415; 0.271]
Gender	− 4.152***	0.612	[− 5.354; − 2.950]	3.988***	1.050	[1.926; 6.049]	1.453	1.083	[− 0.674; 3.579]
Student	1.620	1.100	[− 0.539; 3.778]	− 0.664	1.831	[− 4.258; 2.930]	0.325	1.946	[− 3.496; 4.145]
Employed	0.852	1.162	[− 1.429; 3.132]	− 0.918	1.931	[− 4.710; 2.874]	− 0.398	2.055	[− 4.434; 3.637]
Educational level > 13 years	1.102	0.604	[− 0.084; 2.288]	0.051	1.006	[− 1.924; 2.027]	0.724	1.069	[− 1.374; 2.823]
Marital status	− 0.528	0.851	[− 2.198; 1.143]	0.385	1.414	[− 2.391; 3.162]	0.063	1.505	[− 2.892; 3.019]
Self-reported BMI	− 0.010	0.084	[− 0.176; 0.155]	0.076	0.140	[− 0.199; 0.352]	0.070	0.149	[− 0.223; 0.363]
Tobacco use	0.387	0.539	[− 0.671; 1.444]	0.187	0.896	[− 1.571; 1.945]	0.423	0.953	[− 1.448; 2.294]
CAGE ≥ 2	2.515**	0.800	[0.945; 4.085]	1.495	1.339	[− 1.133; 4.123]	3.030*	1.415	[0.252; 5.809]
Substances use	2.226**	0.683	[0.885; 3.568]	0.454	1.144	[− 1.792; 2.700]	1.813	1.209	[− 0.561; 4.187]
BSI cut-off	5.024***	0.664	[3.720; 6.328]	4.122***	1.148	[1.869; 6.375]	7.190***	1.175	[4.882; 9.497]
Compilation modality	− 0.209	0.619	[− 1.425; 1.006]	1.456	1.029	[− 0.565; 3.476]	1.328	1.096	[− 0.823; 3.479]
*R*^2^ = 0.18; *F*_13; 707_ = 12.29; *p* < 0.001				*R*^2^ = 0.21; *F*_14; 706_ = 13.61; *p* < 0.001			*R*^2^ = 0.11; *F*_13; 707_ = 6.45; *p* < 0.001		

Coding systems: gender: 1 = male 2 = female. Student: 0 = no 1 = yes. Employed: 0 = no 1 = yes. Educational level > 13 years: 0 = no 1 = yes. Marital status: 1 = unmarried 2 = married or living with partner. Tobacco use = 0 = no 1 = yes. CAGE ≥ 2: 0 = no 1 = yes. Illicit drug use: 0 = no 1 = yes. BSI cut-off: 0 = no 1 = yes. Compilation modality: 1 = paper/pencil questionnaires 2 = online questionnaires

Bold values represent* p *< 0.05

Original, *BSMAS* Bergen Social Media Addiction Scale, *MDDI* Muscle Dysmorphic Disorder Inventory, *BMI* body mass index, *CAGE* Cut-Annoyed-Guilty-Eye (CAGE) questionnaire, *BSI* Brief Symptom Inventory

*p < 0.05; **p < 0.01; ***p < 0.001

## Discussion

The present cross-sectional study on 721 young adults showed that (i) SMA-related symptoms were significantly associated with both MD- and ED-related symptoms, (ii) the relationship between symptoms of SMA and EDs was mediated by the severity of MD-related symptoms, with relevant confounding factors (sex, age, educational level, marital status, job status, BMI, problematic alcohol use, tobacco use, illicit drug use, psychopathological distress, compilation modality) being controlled for (Table 4; Fig. 1). In addition to such primary findings related to the mediational model, the study provided evidence suggesting that the severity of SMA-related symptoms was significantly directly correlated with overall psychopathological distress and alcohol misuse severity, and significantly inversely correlated with age and BMI (Table 2).

At the clinical level, the present findings contribute to raise awareness on the risks for mental health related to excessive use of SM [58]. According to established cut-off scores (described above) in the assessment measures, in the present sample there were 154 subjects (21.4%) who met the criteria for PSMU. Among such individuals, there was, compared to subjects without PSMU (Table 3), a significantly higher proportion of females, of students, of subjects with lower educational level, of younger people, and of unemployed individuals; further, there were lower mean BMI values, more severe MD-related symptoms, and a significantly higher proportion of subjects with clinically relevant psychopathological distress and ED-related symptoms. Subsequently, it is possible that people who experience high degree of SMA (with symptoms related to salience, mood modification, tolerance, withdrawal, conflict, and relapse [35]) may take advantage of specifically tailored interventions focused on the prevention of ED-, MD-, and BDD-related disturbances. Such interventions could potentially include SM-based approaches, as studies have suggested that, in certain circumstances, SM platforms can facilitate social interactions, peer support, and access to care, and can be used to promote mental health awareness, thus providing possible benefits in relation to mental disorders [59, 60]. Further, our results suggest that dysfunctional SM use can be relevant in the etiology and/or maintenance of ED- and MD-related disturbances, and subsequently clinicians should consider to assess and treat (see for example [61]) SMA-related symptoms in individuals with such conditions.

In relation to MD, the present study suggests that preoccupation over body image and physical activity, with symptoms related to drive for size (e.g., thoughts of being thinner, desire to increase size and strength), appearance intolerance (e.g., negative beliefs about the body), and functional impairment (e.g., excessive and compulsive exercise) [42], is associated with a range of disturbances related to EDs SMA, and psychopathological distress (Tables 2, 4; Fig. 1). Such findings contribute to elucidate the potential risks associated with maladaptive approaches towards sport and exercise. Physical activity is considered as beneficial for health and mental health [62, 63]; however, in the context of disturbances related to body image such as EDs, BDD and MD, exercising may in some cases be part of dysfunctional attitudes and behaviors, and it is, thus, relevant that clinicians as well as people in the field (e.g., athletes, coaches, gym users, trainers) are aware of such potential harms.

### What is already known on this subject?

These results are consistent with evidence suggesting that excessive exposure to SM is associated with ED pathology, and that the preoccupation over physical appearance and muscularity, which can be triggered by certain SM contents (e.g., “thinspiration” and “fitspiration” posts), plays a relevant role in such phenomenon [5, 18–23, 25].

### What this study adds?

The present cross-sectional study on young adults showed (i) that SMA-related symptoms were significantly associated with both MD- and ED-related symptoms, and (ii) that the relationship between symptoms of SMA and EDs was mediated by the severity of MD-related symptoms, with relevant confounding factors being controlled for (Table 4; Fig. 1).

Such findings add specificity to this field of research as the data of the present research have been collected through quantitative measures, while several previous studies on the topic used qualitative approaches. Further, the present research was specifically focused on psychopathological underpinnings related to SMA, and not to PUI in general. This is especially relevant as within the PUI construc, there are a wide range of phenomena markedly different from SM use, such as video gaming, pornography, cybersex, and online gambling [4]. Consistently with such heterogeneity, while in the present study, it was showed that symptoms related to SMA are related to preoccupation over physical appearance/muscularity, ED-related disturbances, and low BMI, previous studies suggested that other aspects of PUI (e.g., videogaming) can in some cases facilitate sedentarism, use of junk food and increased BMI [5, 64]. Thus, given the design of the study, the study findings are specific for SMA, and do not apply to different types of PUI.

### Strength and limits

The present study has several limitations, among which: (i) despite we used an hypothesis-driven statistical approach specifically designed to infer causal relationships between associated variables, definitive causality cannot be established as a complete study of mediation processes would require a longitudinal approach, while we used a cross-sectional approach; (ii) self-reports, non-validated by a clinical interview, have been used, whose results may be affected by several biases (e.g., social desirability bias, response bias) [65]; (iii) we did not collect data on the psychiatric history of participants, although the research was specifically directed towards a general population sample and psychopathology was assessed through self-report measures; (iv) a selection bias of the sample may have occurred, i.e., questionnaires might have been more accessible to certain groups of individuals (e.g., students compared to employed or unemployed people, females compared to males); (v) although we assessed the severity of symptoms related to SMA (related to the domains of salience, mood modification, tolerance, withdrawal, conflict, and relapse), we did not perform a qualitative analysis of the SM contents accessed by users; (vi) the amount of subjects who met the criteria for PSMU (21.4%), clinically relevant ED-related symptoms (16.6%), clinically relevant MD (3.6%), clinically relevant psychopathological distress (23.6%), and PAU (13.0%) can be influenced by the fact that the majority of individuals (63.1%) were recruited during the COVID-19 outbreak, which is a risk factor for increased psychopathology and addiction-related problems [53–55], although the compilation timing (i.e., November 2019–March 2020 vs September–December 2020) was included as a covariate in the mediational model; (vii) although sex was included as a covariate in the mediational model, it is relevant to observe that MD-related symptoms are more frequently observed in males than in females [66], while our sample included more females (69.9%) than males (30.1%). Among the strengths of the study: (i) this is, to the best of our knowledge, the first research specifically aimed at investigating in young adults the mediating role of MD-related symptoms on the relationship between SMA- and ED-related symptoms; (ii) the sample size (*n* = 721) was adequate, as indicated by a priori power analysis; (iii) we used extensively validated assessment instruments; (iv) we statistically controlled for potential confounders.

## Conclusions

In conclusions, our findings provide pieces of insight on the complex relationships between SMA, MD, and EDs. It is possible that, while SM use is not detrimental for mental health per se and it can even be integrated in telepsychiatry approaches [59, 60], a dysfunctional use of SM can expose users to unrealistic body image ideals; this can have a role in worsening emotional wellbeing and in favoring a maladaptive approach towards physical exercise and food habits, thus contributing to induce in vulnerable subjects symptoms related to MD and EDs. Given the diffusion of SM use among young people, mental health preventive interventions should be encouraged in relation to such phenomena.

## Data Availability

The data that support the findings of this study are available from the corresponding author upon reasonable request.
